# Sinister Self-Sacrifice: The Contribution of Apoptosis to Malignancy

**DOI:** 10.3389/fimmu.2014.00299

**Published:** 2014-07-04

**Authors:** Jorine J. L. P. Willems, Benjamin P. Arnold, Christopher D. Gregory

**Affiliations:** ^1^MRC Centre for Inflammation Research, Queen’s Medical Research Institute, The University of Edinburgh, Edinburgh, UK

**Keywords:** apoptosis, inflammation, lactoferrin, phagocytosis, tumor-associated macrophages

Induction of apoptosis is one of the main defenses of the body against cells that have acquired malicious mutations. It may seem counter-intuitive then, that massive cell death is observed in many malignant tumors (1, 2). Despite high rates of apoptosis, these tumors continue to grow rapidly. Thus, tumor cell growth must outbalance tumor cell death. Intuitively presumed only to inhibit tumor growth, apoptotic cells may actually promote net tumor growth (3, 4). As long ago as 1956, Revesz showed that cell death can enhance tumor growth (5). Moreover, new studies in progress in our laboratory show that apoptosis in tumor cells promotes growth rates in aggressive B-cell lymphoma.

Cells undergoing apoptosis are difficult to observe *in vivo*, as they are rapidly cleared by phagocytosis, most obviously by macrophages. Accumulation of macrophages, sometimes engorged with apoptotic cells, is observed in many malignant tumors and is generally associated with poor prognosis (6, 7). Inflammatory cells, in particular macrophages, are key elements of the tumor environment, providing support for the continually expanding “rogue” tissue. The tumor microenvironment resembles that of a wound that fails to heal (8), where macrophages not only clear and repair, but also promote tissue regeneration and support. Tumor-associated macrophages (TAM) display a phenotype that is reminiscent of wound-healing macrophages. They have been shown to promote angiogenesis, tissue remodeling, and anti-inflammatory responses, which results in the support of tumor cell growth and metastasis (9–12).

Apoptosis of tumor cells in a growing malignant tissue may therefore be rationalized as a “sinister sacrifice” of some cancer cells that ultimately facilitates cancer progression. We hypothesize that apoptotic cells play a key role in driving oncogenesis, both through the release of soluble and microvesicle-associated signaling factors, as well as through direct interaction with phagocytes. Here we postulate lactoferrin (Lf) as an important signaling factor maintaining an anti-inflammatory tumor microenvironment, and stress the importance of apoptotic cell engulfment by macrophages for driving a pro-tumor phenotype in TAM (Figure 1).

**Figure 1:**
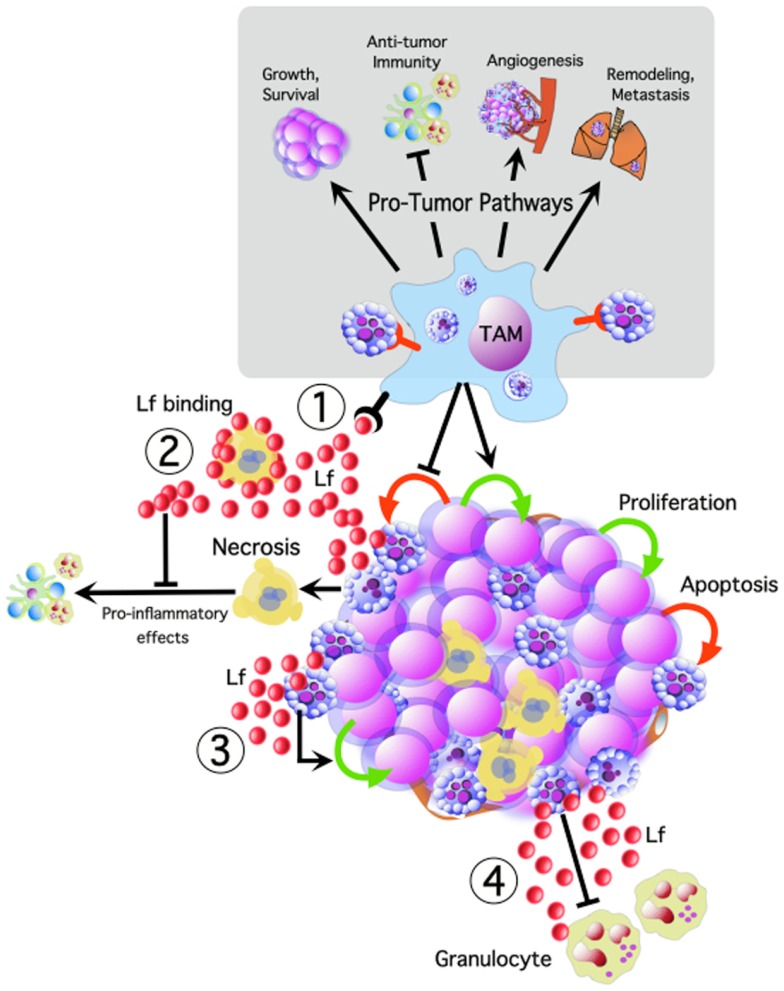
**Tumor cell apoptosis potentially activates multiple oncogenic pathways, promoting tumor cell growth and survival, angiogenesis, remodeling, and metastasis, while inhibiting anti-tumor immune responses**. We propose that TAM interacting with apoptotic tumor cells are central to many of these pathways. Apoptotic cells release lactoferrin (Lf) which could promote tumor growth and progression by: (1) TAM activation, (2) modulating the inflammatory effects of necrosis, (3) acting as a direct trophic factor, and (4) functioning as a “keep-out” signal to anti-tumor granulocytes.

## Direct Effects of Lactoferrin

Cells undergoing apoptosis release a variety of biologically active “find-me” or “keep-out” signaling factors, including the nucleotides ATP and UTP (13, 14), the lipid lysophosphatidylcholine (LPC) (15), as well as the proteins fractalkine (16), Lf (17), and monocyte chemotactic protein (MCP-1) (18). Some of these signaling molecules may be associated with microvesicles, as is the case with the chemokine, fractalkine (16), which may support prolonged biological activity. It has been hypothesized that these find-me signals not only affect chemotaxis and phagocytosis, but may also have additional pleiotropic biological effects. Indeed, ATP released by apoptotic cells increased binding of apoptotic cells to macrophages (19). Furthermore, fractalkine has been shown to stimulate pro-survival and growth-promoting effects (20, 21) and was found to cause the expression of milk fat globule epidermal growth factor (MFG-E8) on macrophages, which leads to enhanced apoptotic cell clearance (22).

Lactoferrin was identified in our laboratory to be released from apoptotic cells. Lf is produced *de novo* by a diverse range of cells stimulated to undergo apoptosis *in vitro* (17). This 80 kDa iron-binding glycoprotein is well-documented to have immunomodulatory, antimicrobial, anti-inflammatory, and trophic activities (23–26). We propose that Lf is another pleiotropic molecule released from apoptotic cells that can regulate the tumor microenvironment. Thus, since it is well-known that apoptosis is frequent in several types of cancer, particularly high-grade forms (27), it is conceivable that persistence of uncleared apoptotic cells (which may occur through saturation) could enable these cells to become secondarily necrotic with the potential consequences of release of noxious contents via cell lysis leading to activation of pro-inflammatory responses (28). However, most malignant tumors maintain a phenotype that militates against anti-tumor immune and inflammatory responses. Given our previous findings that Lf is released from cells undergoing apoptosis (17), together with our unpublished studies showing that Lf binds to necrotic cells, we suggest that Lf serves to dampen down pro-inflammatory responses resulting from persistent secondarily necrotic cells. In fact, it has been shown that necrotic neutrophil lysates, which contain large quantities of Lf from the secondary granules, are anti-inflammatory, and are able to inhibit the production of pro-inflammatory cytokines, such as tumor necrosis factor-α (TNFα), IL6, IL8, and IL1β, by macrophages (29). The mechanism through which this is achieved may involve the “mopping up” of necrotic cell-released pro-inflammatory contents by Lf.

Lactoferrin has a highly positively charged N-terminal end (30), which is capable of interacting with a variety of proteins and membranes, but can also bind a selection of metal ions as well as iron (31). Furthermore, Lf can interact with lipid A of lipopolysaccharide (LPS) causing the neutralization of LPS-stimulated secretion of pro-inflammatory cytokines by monocytic cells, including TNFα, IL1β, IL6, and IL8 (32, 33). Mopping of noxious contents by Lf may be a final safeguard system to prevent pro-inflammatory responses at sites of high rates of apoptosis. This may not only help maintain the anti-inflammatory environment in tumors, but could also play a role in the resolution of inflammation, where neutrophil activation and death may lead to the release of large quantities of Lf. In addition, in tumors characterized by neutrophil infiltration, the dominant source of biologically active Lf may be derived from neutrophils, rather than apoptotic tumor cells.

Lactoferrin is also known to directly exert anti-inflammatory effects by inhibiting the migration of neutrophils (17) and also by indirectly enhancing the production of anti-inflammatory cytokines including IL4, IL10, and transforming growth factor-β (TGFβ) (25, 26). Some studies also suggest that Lf can directly interact in the nuclear factor κB (NFκB) pathway interfering with its binding to DNA (33).

These findings point to a possible direct mechanism of Lf for controlling pro- and anti-inflammatory cytokine expression. In high-grade malignancies, these effects of Lf could help moderate anti-tumor inflammatory and immune responses, allowing continued malignant growth. The pro-tumor effects of Lf are likely to be context dependent, however, since Lf has been shown to have pro-inflammatory, immunostimulatory, and cell growth-inhibitory effects (34–36) as well as anti-inflammatory and trophic properties. An open, and important question is whether Lf is released by dying tumor cells as a consequence of anti-tumor therapy and, if so, whether it has properties which could ultimately confound – or alternatively facilitate – long-term therapeutic efficacy. Again, the significance of Lf may be tissue context dependent.

## Effects of Phagocytes Interacting with Apoptotic Tumor Cells

In addition to the release of signaling factors, interaction of apoptotic cells with phagocytes also provides opportunities for regulating tumor cell growth. TAM are the most important phagocytes of apoptotic tumor cells in most cancers, and often prominently display engulfed remnants of apoptotic cells (2, 37, 38). Current work in our laboratory indicates that the TAM of aggressive B-cell lymphoma show up-regulated expression of receptors involved in the recognition and engulfment of apoptotic cells. Furthermore, recent studies in mice have shown that radiotherapy, one of the most important anti-cancer treatment strategies, can enhance tumor cell repopulation *in vivo*, through the induction of apoptosis (4). Such effects may be mediated via responses of macrophages that accumulate as a result of the massive radiation-induced apoptosis as previously proposed (39). Apart from preventing the build-up of free apoptotic cells, removal of apoptotic cells by phagocytosis may therefore drive the pro-tumor activation status of TAM.

Engulfment of apoptotic cells by macrophages has been found to activate downstream signaling pathways that cause the up-regulation and secretion of anti-inflammatory mediators such as IL10, and TGFβ, and the down-regulation of pro-inflammatory mediators such as IL6, IL8, IL12, and TNFα (40–43). Furthermore, incubation of phagocytes with apoptotic cells reduces the effects of LPS, increasing release of IL10, while reducing TNFα, IL1β, and IL12 release. Blocking apoptotic cell engulfment can prevent these responses (44). As well as enhancing anti-inflammatory effects, apoptotic cells have also been shown to promote tumor growth and angiogenesis. Phagocytes can release growth factors upon engulfment of apoptotic cells, including VEGF (45), and apoptotic cells can induce angiogenesis via electrostatic effects (46).

Given the abilities of apoptotic cells to induce anti-inflammatory signaling, angiogenesis, and the release of growth factors by TAM, it will be important to determine to what extent they influence additional pro-tumor macrophage properties such as matrix remodeling, invasion, and metastasis.

## Conclusion

We propose that the apoptotic cell contributes markedly to the conditioning of the tumor microenvironment. Here, we suggest that Lf released from apoptotic cells could contribute to the anti-inflammatory state of the tumor microenvironment. Furthermore, engagement of apoptotic cells by macrophages may also inhibit anti-tumor inflammatory and immune responses, as well as promote tumor cell growth, angiogenesis, and tissue remodeling. These normal, physiological effects of apoptosis endow this fundamental cell death process with regulated and homeostatic properties that permit tissue turnover, organogenesis, and wound healing. However, these properties may be hijacked in malignant disease in order to facilitate cancer progression.

Understanding the complexity of the signaling of apoptotic tumor cells to viable tumor cells, macrophages, and other elements of the tumor environment will be key to improving tumor treatment outcomes and to prevent metastasis, by targeting the interaction of the host with apoptotic cancer cells. This is especially important since most anti-cancer therapies are designed to induce apoptosis of malignant cells, which, without inhibition of these interactions, could ultimately facilitate tumor repopulation.

## Conflict of Interest Statement

The authors declare that the research was conducted in the absence of any commercial or financial relationships that could be construed as a potential conflict of interest.
